# IL-6-induced epithelial-mesenchymal transition promotes the generation of breast cancer stem-like cells analogous to mammosphere cultures

**DOI:** 10.3892/ijo.2011.1275

**Published:** 2011-11-30

**Authors:** GUOZHU XIE, QIWEI YAO, YING LIU, SHASHA DU, AIHUA LIU, ZHAOZE GUO, AIMIN SUN, JIAN RUAN, LONGHUA CHEN, CHANGSHENG YE, YAWEI YUAN

**Affiliations:** 1 Department of Radiation Oncology, Nanfang Hospital, Southern Medical University, Guangzhou, Guangdong 510515, P.R. China; 2 Department of Respiratory Disease, Nanfang Hospital, Southern Medical University, Guangzhou, Guangdong 510515, P.R. China; 3 Department of Breast Center, Nanfang Hospital, Southern Medical University, Guangzhou, Guangdong 510515, P.R. China; 4 Research Center of Clinical Medicine, Nanfang Hospital, Southern Medical University, Guangzhou, Guangdong 510515, P.R. China

**Keywords:** breast cancer, stem cells, epithelial-mesenchymal transition, IL-6, mammosphere culture

## Abstract

Recently, the inflammatory cytokine IL-6 has been reported as a potent inducer of epithelial-mesenchymal transition (EMT) in breast cancer cells with an epithelial phenotype. Furthermore, EMT induces stem cell features in normal and transformed mammary cells. We explored whether IL-6-induced EMT promoted the generation of breast cancer stem-like cells (BrCSCs) in epithelial-like breast cancer cells, and whether the cytokines EGF and bFGF, analogous to IL-6, *per se* induced epithelial-mesenchymal transition, resulting in the enrichment of BrCSCs in mammosphere cultures. Herein, we provide evidence that IL-6 is capable of generating CD44^+^ cells with stem-like properties through induction of the EMT in the epithelial-like T47D breast cancer cells. We also show that mammosphere cultures of epithelial-like breast cancer cells, T47D, MCF7, ZR-75-1 and MDA-MB-453 cells, consistently generated stem-like cancer cells solely as a result of the EGF and bFGF cytokines in the mammosphere media mediating EMT. This finding demonstrated the link between the inflammatory cytokine IL-6 and BrCSCs and identified an important mechanism for the enrichment of BrCSCs in mammosphere cultures. Thus, EMT appears to be a critical mechanism for the induction of cancer cells with stem-like properties, and EMT of non-stem cancer cells could be a source of CSCs.

## Introduction

Interleukin-6 (IL-6) is a pleiotropic cytokine that plays an important role in many chronic inflammatory diseases. Recent studies have shown that IL-6 plays a primary role in the pathophysiology of cancer (1,2). In breast cancer, tumor tissue exhibits high expression levels of IL-6 compared with matched normal breast tissue samples, which also correlate with more advanced tumor grades (3). Furthermore, elevated serum IL-6 levels correlate with advanced breast tumor stage (4), increased number of metastatic sites (4), and poor survival in patients with breast cancer (5).

Cancer stem-like cells (CSCs) are a highly tumorigenic cell type. CSCs exist as a small subset within tumors and are hypothesized to be critical initiators of cancers, as well as sustaining tumor growth and producing metastases (6–8). They also mediate resistance to conventional anti-tumor therapies (9). Breast cancer is the first human tumor for which a putative CSC subpopulation has been isolated as CD44^+^CD24^−/low^ cells (10). These breast CSCs (BrCSCs) exhibit high tumorigenicity when injected into immunocompromized mice, and possess characteristics that are associated with normal stem cells; specifically, they have the ability to give rise to all cell types found in a particular cancer sample.

In view of the important role of IL-6 in the malignant features of cancer, we were interested to explore the relationship between IL-6 and BrCSCs. Recent studies have indicated that IL-6 is capable of inducing an epithelial-mesenchymal transition (EMT) phenotype in human breast cancer cells (11). The induction of EMT in immortalized human mammary epithelial cells (HMLEs) results in the generation of cells with stem cell properties (12,13). We hypothesized that IL-6 promotes the generation of cancer cells with stem-like properties by induction of EMT. We also proposed that the mammosphere culture is able to enrich CSCs from tumor samples as a result of cytokines in the mammosphere media inducing EMT in cancer cells, in a similar manner to IL-6.

Herein, we provide evidence that the inflammatory cytokine, IL-6, is capable of generating CD44^+^ cells with stem-like properties through inducing EMT in the T47D breast cancer cells. We also show that mammosphere culture consistently generated stem-like cancer cells solely as a result of the EGF and bFGF cytokines in the mammosphere media mediating EMT. Thus, EMT appears to be an important mechanism for the induction of cancer cells with stem-like properties, and EMT of non-stem cancer cells could be a source of CSCs.

## Materials and methods

### Cell culture

Human breast cancer cell lines, including T47D, MCF7, ZR-75-1, and MDA-MB-453, were obtained from the Cell Bank of Type Culture Collection of Chinese Academy of Sciences (Shanghai, China). T47D, ZR-75-1, and MDA-MB-453 cells were routinely maintained in RPMI-1640 medium supplemented with 10% fetal bovine serum (FBS; Hyclone, Logan, UT, USA). MCF7 cells were cultured in DMEM medium containing 10% FBS. IL-6 (Peprotech, Rocky Hill, NJ, USA) was added to all cultures at final concentrations of 50 ng/ml in RPMI-1640 containing 10% FBS.

For the mammosphere culture, cells were suspended at 50,000 cells/ml in DMEM/F12 (1:1) containing 5 μg/ml bovine insulin (Sigma, St. Louis, MO, USA), 0.4% bovine serum albumin (BSA; Sigma), 2% B27 (Invitrogen, Carlsbad, CA, USA), 20 ng/ml basic fibroblast growth factor (bFGF; Peprotech) and 20 ng/ml epidermal growth factor (EGF; Sigma); cells were then seeded into 6-well plates (3 ml per well). As they differentiated during culture, cells were reseeded to standard adherent culture conditions in serum-supplemented media.

### Flow cytometric analysis

Cells were trypsinized, suspended into single-cell mixtures, washed with phosphate buffered saline (PBS), and incubated on ice for 30 min with monoclonal antibodies specific for human cell surface markers CD44-FITC (eBioscience, San Diego, CA, USA) or CD24-PE (eBioscience). In negative control experiments, cells were incubated with fluorescence-labeled isotype-matched pre-immune IgG instead. Cells were washed and analyzed using a flow cytometer (BD FACS Aria, San Jose, CA, USA).

### Real-time quantitative PCR

Cells were harvested, and RNA was extracted using TRIzol (Invitrogen) following the manufacturer's protocol. One microgram of total RNA was reverse transcribed into cDNA using the SuperScript First-Strand Synthesis System (Invitrogen). Real-time polymerase chain reactions (PCRs) using the SYBR Green PCR Master Mix were performed using an ABI PRISM 7500 Sequence Detection System (Perkin-Elmer/Applied Biosystems, Rotkreuz, Switzerland). Data are shown after normalization to 18S expression. Primer sequences were as follows: E-cadherin, forward, 5′-CCCACCACGTAC AAGGGTC-3′, reverse, 5′-CTGGGGTATTGGGGGCATC-3′; vimentin, forward, 5′-CGCCAGATGCGTGAAATGG-3′, reverse, 5′-ACCAGAGGGAGTGAATCCAGA-3′; twist, forward, 5′-GTCCGCAGTCTTACGAGGAG-3′, reverse, 5′-GCTTGAGGGTCTGAATCTTGCT-3′; CD44: forward, 5′-CAGCAACCCTACTGATGATGACG-3′, reverse, 5′-GCCA AG AGGGATGCCAAGATGA-3′; 18S, forward, 5′-CCTGGA TACCGCAGCTAGGA-3′ reverse, 5′-GCGGCGCAATA CGAATGCCCC-3′.

### Mouse injections

Animal maintenance and experiments were performed in accordance with the animal care guidelines of the Southern Medical University, Guangzhou, China. Cells were resuspended in a 1:1 (v/v) mixture of culture media and Matrigel (BD Biosciences, San Jose, CA, USA), and 10^6^ CD44^+^ or CD44^−^ cells were injected subcutaneously into the mammary fat pads of 4-week old female NOD/SCID mice. Tumor growth was monitored twice a week with callipers at the site of injection for 40 days. Animals were sacrificed as soon as tumor size reached 1.0 cm in diameter.

### Invasion assay

Transwell insert chambers with an 8-μm porous membrane (Corning Costar, Cambridge, MA, USA) were used for the assay. Transwell insert chambers were pre-coated with a 1:5 (v/v) mixture of Matrigel (BD Biosciences) and RPMI-1640 medium. The following day, cells were washed three times with PBS and 1×10^5^ cells were added to the top chamber in serum-free media. The bottom chamber was filled with media containing 10% FBS. Cells were incubated for 24 h at 37°C in a 5% CO_2_ humidified incubator. To quantify the number of invasive cells, cells on the top chamber were removed with a cotton-tipped swab, and migrated cells were fixed in methanol and stained with 1% crystal violet. Five random fields were counted.

### Western blot analysis

Primary antibodies included mouse anti-E-cadherin (1:5,000; BD Biosciences), mouse anti-vimentin (1:500; Clone V9, Dako, Glostrup, Denmark), and rabbit anti-CD44 (1:5,000; GeneTex Inc., Irvine, CA, USA). Secondary antibodies included rabbit anti-mouse IgG-HRP (1:1,000; Santa Cruz Biotechnology, Santa Cruz, CA, USA) and goat anti-rabbit IgG-HRP (1:1,000; GE Healthcare, Chalfont St Giles, UK). HRP-conjugated monoclonal mouse anti-GAPDH (Kangchen, Shanghai, China) was used as an internal parameter. All antibodies were diluted with 5% milk in PBS containing 0.1% Tween-20 (PBS-T) and incubated for either 1 h at room temperature or overnight at 4°C. All Western blots were visualized with ECL Western blotting substrate (Pierce, Rockford, IL, USA).

### Immunofluorescence

A total of 1×10^5^ cells per chamber were plated into Lab-Tek two-chamber slides overnight. The next day, when cells were 50–70% confluent, they were washed with PBS twice, fixed in 3% paraformaldehyde (Sigma) and permeabilized in 0.1% Triton X-100 (Sigma) in PBS buffer at 4°C for 30 min. The cells were then washed 3 times with PBS and incubated with blocking solution (10% horse serum in PBS). The cells were then incubated with primary antibodies for anti-E-cadherin (BD Biosciences) or anti-vimentin V9 (Clone V9, Dako) overnight at 4°C. The cells were washed three times in PBS and incubated with the secondary antibody, goat anti-mouse-Alexa Fluor 488 (1:1,000; Molecular Probes, Invitrogen) in blocking buffer for 1 h at room temperature in the dark. Finally, the cells were washed three times in PBS and incubated with 0.25 mg/ml DAPI (Roche) for 1 min at room temperature in the dark. The slides were washed extensively with PBS and mounted with Fluoromount-G (Southern Biotech, Birmingham, AL, USA). All matched samples were photographed (controls and tests) using immunofluorescence microscope (Olympus BX51, Tokyo, Japan) with identical exposure times.

### Irradiation and clonogenic assay

Cells were dissociated by trypsinization and mechanical agitation with a Pasteur pipette into single cell suspensions in RPMI-1640 medium supplemented with 10% FBS. The cells were seeded in 6-well plates at the indicated densities, and then incubated overnight before the irradiation treatment. Cells were irradiated from a vertical direction at a dose rate of 400 cGy/min with 6-MV X-rays produced by a Varian 2100C linear accelerator at the Southern Medical University. Cells were irradiated for the time required to receive a total dose of 0, 2, 4, 6, or 8 Gy. Negative control cells were sham-irradiated. Following the irradiation, the cells were incubated for 15 days at 37°C in a 5% CO_2_ environment to allow the formation of colonies. The resulting colonies were fixed with 100% ethanol and stained with 1% crystal violet. Colonies containing >50 cells were counted as clonogenic survivors. Three independent experiments were performed, each in triplicate. The surviving fraction was calculated as described previously (14). Using GraphPad Prism 5 software (GraphPad, La Jolla, CA, USA), the data were fitted into the following single-hit multitarget formula: S=1-(1-e-D/D0)N.

### Assessment of proliferation and doxorubicin resistance

Cell proliferation potential and the relative resistance to doxorubicin were evaluated by cell proliferation assays using a Cell Counting Kit-8 (CCK8, Yiyuan Biotechnologies, Guangzhou, China). Cells were plated at a concentration of 1×10^3^ cells per well (for growth advantage assays) or 1×10^4^ cells per well (for doxorubicin resistance) into 96-well culture plates. For the cell proliferation potential assay, 10 μl CCK-8 solution was added on days 1–5. For the doxorubicin resistance assay, 10 μl of CCK-8 solution was added to each well of the plate 4 h, and 1–5 days after treatment with doxorubicin at a final concentration of 10 μg/ml. After the addition of CCK-8 solution, plates were incubated for 4 h, and absorbance was then measured at 450 nm using a microplate reader (SpectraMax M5, Sunnyvale, CA, USA).

### Statistical analysis

In all experiments, differences among groups were analyzed by ANOVA or Student's t-test using SPSS version 13.0 (SPSS, Chicago, IL, USA). A p=0.05 was considered to be statistically significant.

## Results

### The enrichment of CD44-positive cells by IL-6 exposure

**Figure 1:**
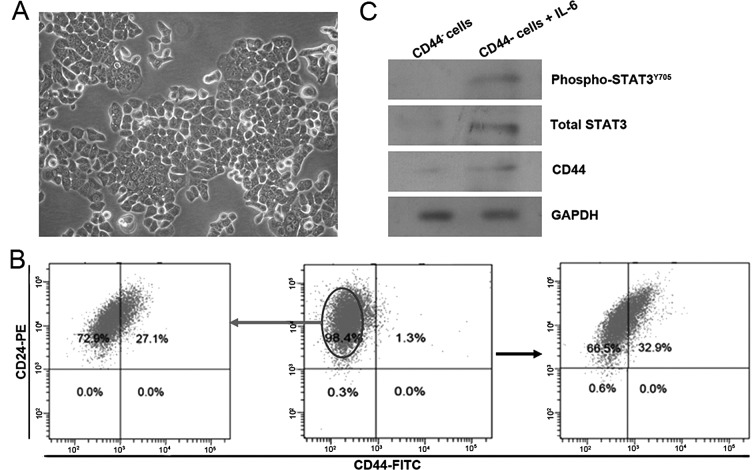
(A) T47D cells exhibit epithelial-like features and a cobblestone-like morphology. (B) T47D cells contained very low proportions of CD44^+^ cells (middle); the number of CD44^+^ cells are increased by ~30 times after 10 days of 50 ng/ml IL-6 exposure (right); CD44^−^ cells isolated from T47D cells showed a similar enrichment of CD44^+^ cells after IL-6 exposure (left). (C) Western blot analysis showed IL-6-treated CD44^−^ cells expressed elevated active STAT3^Y705^ (phospho-STAT3^Y705^), total STAT3, and CD44.

Human breast cancer T47D cells, which are characterized as estrogen and progesterone receptor (ER/PR) positive and Her-2/neu (Her2) negative, were cultured in RPMI-1640 medium containing 10% FBS. T47D cells exhibit epithelial-like features (Fig. 1A), and contain a very low proportion of CD44^+^ cells (Fig. 1B middle). After 10 days of exposure to 50 ng/ml IL-6, the number of CD44^+^ cells had increased by ~30-fold, as shown by the FACS analysis (Fig. 1B right). To determine whether this enrichment of CD44^+^ cells was because CD44^+^ cells that already existed in T47D cell cultures prior to the exposure to IL-6 that acquired a growth advantage in IL-6-containing media, CD44^−^ cells were isolated from T47D cells and cultured in IL-6-containing media. The results showed a similarly significant enrichment of CD44^+^ cells (Fig. 1B left). Western blot analysis showed IL-6-treated cells expressed elevated active STAT3^Y705^ (phospho-STAT3^Y705^), a main downstream molecular of IL-6 signaling, and total STAT3, which coincided with induction of CD44 (Fig. 1C). These data showed that IL-6 exposure activated IL-6/STAT3 signaling in CD44^−^ T47D cells, and induced up-regulation of CD44 protein expression, resulting to the enrichment of CD44^+^ cell population.

### CD44^+^ cells induced by IL-6 exposure exhibit many properties of CSCs

**Figure 2:**
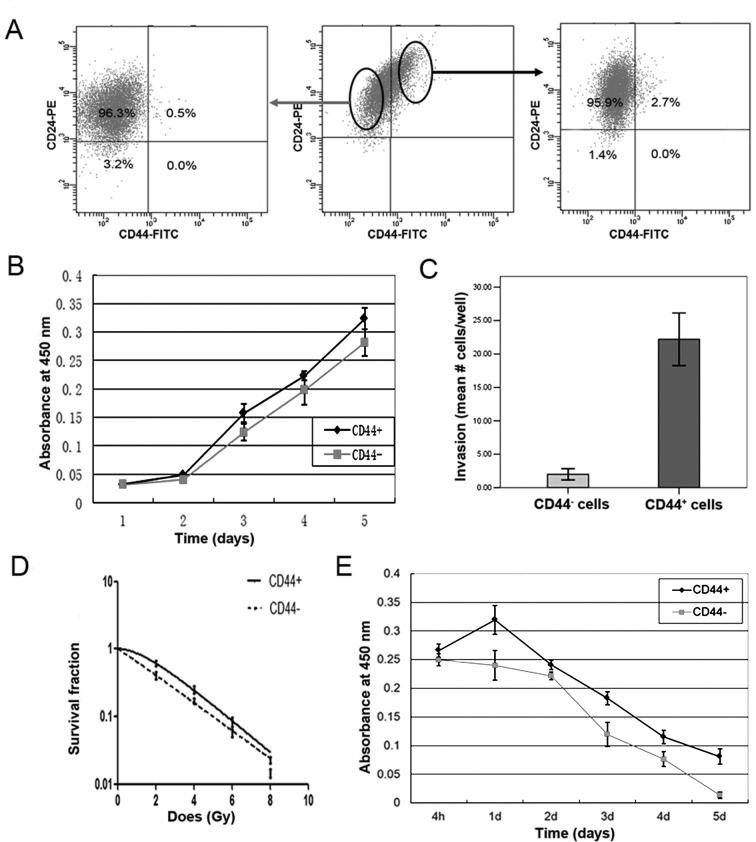
(A) CD44^+^ cells induced by IL-6 differentiated into a variety of cell types after 10 days in standard culture conditions (right); few CD44^+^ cells were generated by culturing the isolated CD44^−^ cells in the standard culture conditions for the same period of time (left). (B-E) IL-6 induced CD44^+^ cells displayed significantly enhanced proliferation (B) and invasive (C) potential, increased resistance to radiation (D), and reduced cell death due to doxorubicin (E), compared with corresponding CD44^−^ cells.

CD44 is an important CSC marker in breast cancer cells, especially in epithelial-like breast cancer cells. We therefore speculated that the CD44^+^ cells resulting from IL-6 exposure might exhibit phenotypes similar to those reported previously for CSCs. To test this hypothesis, IL-6-induced cells were fractionated based on their CD44/CD24 antigen marker profile. Tumorigenic potential was evaluated by injecting CD44^+^ cells and CD44^−^ cells into the mammary fat pads of NOD/SCID mice. As anticipated, the CD44^+^ cells displayed enhanced tumorigenic potential compared with CD44^−^ cells (2/5 for CD44^+^ versus 0/5 for CD44^−^cells). Furthermore, these isolated CD44^+^ cells differentiated into a variety of cell types, as did parental T47D cells, after 10 days of culture in standard serum-supplemented culture conditions (Fig. 2A right). In contrast, no CD44^+^ cells were generated by culturing isolated CD44^−^ cells in standard culture conditions for the same period of time (Fig. 2A left). We confirmed and extended these findings by purifying single-cell clones from CD44^+^ and CD44^−^ cell populations, and observed similar behavior ***in vitro*** (data not shown).

To investigate further whether they exhibited other enhanced malignant features, the proliferation and invasive potential, and the response to radiation and doxorubicin, of CD44^+^ cells induced by IL-6 exposure were determined. The IL-6-induced CD44^+^ cells displayed significantly enhanced proliferation potential (Fig. 2B) and increased invasive potential (Fig. 2C) compared with CD44^−^ cells, as demonstrated by the CCK-8 and transwell assays, respectively. Additionally, the IL-6-induced CD44^+^ cells exhibited increased resistance to radiation (Fig. 2D), and reduced cell death after doxorubicin treatment (Fig. 2E) compared with CD44^−^ cells, which was consistent with the characteristics of breast cancer stem cells (BrCSCs) reported in previous studies (15,16). These data suggest that CD44^+^ cells induced by IL-6 exposure exhibit the characteristics of CSCs.

### CD44^+^ T47D cells induced by IL-6 exposure undergo EMT

**Figure 3:**
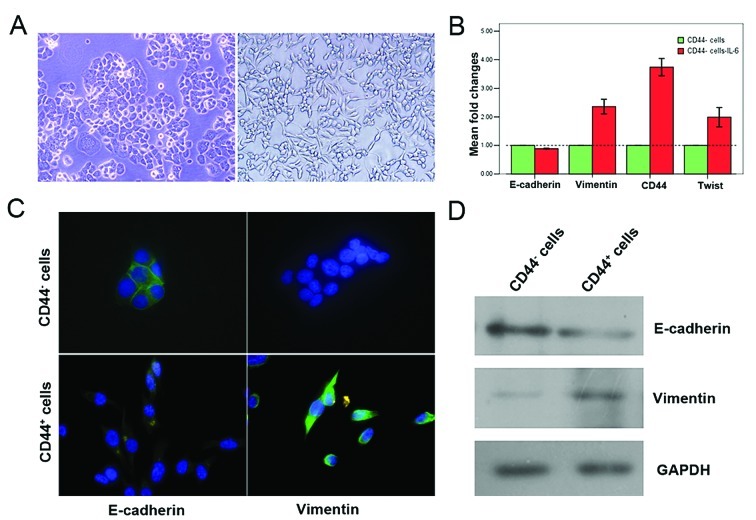
(A) Morphological changes from a cobblestone-like (left) to a spindle-like (right) morphology were seen 10 days after IL-6 exposure. (B) CD44^−^ cells isolated from T47D cells showed a gene expression pattern consistent with EMT after treated with IL-6, as demonstrated by quantitative real-time PCR analysis. (C) Immunofluorescence analysis was utilized to compare expression of E-cadherin and vimentin in CD44^−^ vs. CD44^+^ cells. (D) Western blot analyses showed the down-regulation of E-cadherin expression and the induction of vimentin.

A previous study has shown that IL-6 can induce the EMT phenotype in human breast cancer epithelial-like cell lines (11). To explore whether the enrichment of CD44^+^ cells by exposure to IL-6 is associated with EMT phenotypes, the CD44^−^ sub-population was isolated from T47D cells and subjected to induction with 50 ng/ml IL-6. Morphological changes from a cobblestone to a spindle-like morphology, a classical marker of EMT induction, were seen 10 days after IL-6 exposure (Fig. 3A). Quantitative real-time PCR analysis showed a gene expression pattern that was consistent with EMT, including E-cadherin repression and the concomitant induction of vimentin and twist, which was accompanied by the induction of CD44 (Fig. 3B). Immunofluorescence microscopy was utilized to compare the immunostaining of E-cadherin and vimentin in CD44^−^ versus CD44^+^ cells. CD44^−^ cells showed epithelial homophilic adhesion and prominent levels of E-cadherin, and lacked the expression of vimentin. CD44^+^ cells displayed decreased E-cadherin and prominent vimentin expression (Fig. 3C). Western blot analyses also showed the down-regulation of E-cadherin expression and the induction of vimentin (Fig. 3D).

### CD44^+^ cells induced by IL-6 exposure resemble CD44^+^ cells enriched by mammosphere culture

**Figure 4:**
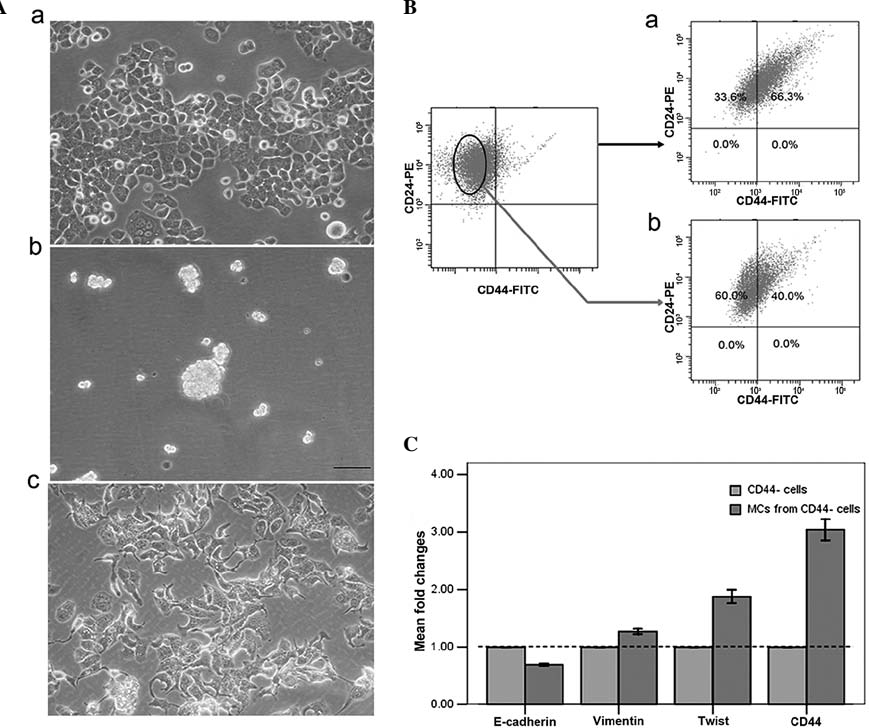

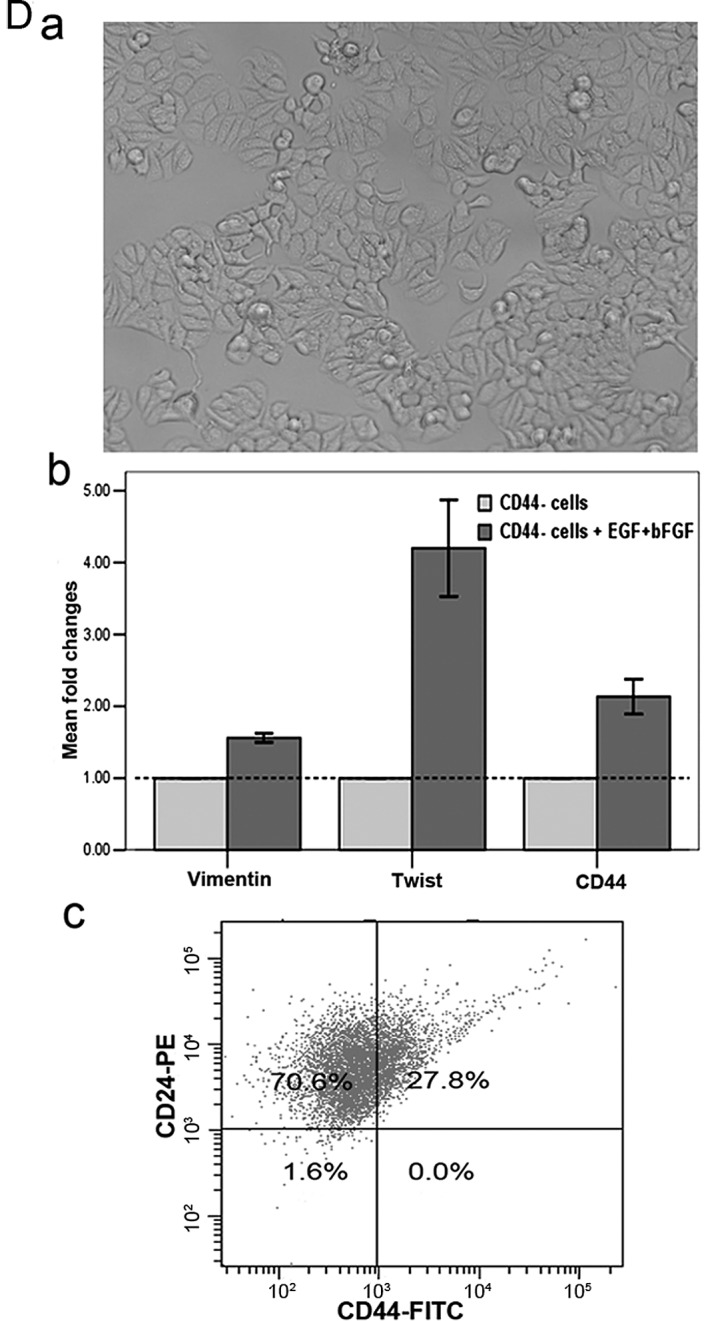
(A) T47D cells were grown as monolayer culture (a); mammosphere culture of T47D cells formed suspended spheres (b); CD44^−^ cells exhibited a spindle-like morphology and mesenchymal appearance after 24-h culture in mammosphere media supplemented with 3% FBS (c). (B) Mammospheres were enriched for CD44^+^ cells but not for CD44^+^CD24^−/low^ cells (a); mammosphere culture of CD44^−^ cells isolated from T47D cell cultures were similarly enriched for CD44^+^ cells (b). (C) After only 12 h, mammosphere cells cultured from CD44^−^ cells consistently downregulated the expression of mRNAs encoding epithelial markers, such as E-cadherin, and upregulated mRNAs encoding mesenchymal markers, such as vimentin and twist. (D) CD44^−^ cells exhibited a spindle-like morphology after 24-h culture in standard media containing EGF and bFGF (a), and up-regulated the expression of mesenchymal markers (vimentin and twist) and CD44 even at 12 h after culture in the media (b); CD44^+^ cells were enriched after a 1-week culture (c).

The non-adherent mammosphere culture system, in which stem-like cells are capable of forming suspended spheres, has been extensively utilized to enrich cultures for BrCSCs with the CD44^+^CD24^−/low^ phenotype (17). Similarly, the mammosphere culture of T47D cells resulted in the formation of suspended spheres (Fig. 4A-b). Interestingly, these formed mammospheres were significantly enriched for CD44^+^ cells but not for CD44^+^CD24^−/low^ cells (Fig. 4B-a). This finding was consistent with the induction of CD44^+^ cells by IL-6 exposure, which were also enriched for CD44^+^ cells but not for CD44^+^CD24^−/low^ cells. These data indicated that IL-6 exposure resembles mammosphere culture, in that it can induce cancer stem-like cells in cultures of T47D cells. We found that the mammosphere culture of CD44^−^ cells isolated from T47D cells identically enriched for CD44^+^ cells (Fig. 4B-b), which was coincident with the result that CD44^+^ cells were induced from CD44^−^ cells in T47D cells by IL-6 exposure. Cells exhibited a spindle-like morphology and mesenchymal appearance (Fig. 4A-c) after 24 h culture in mammosphere media supplemented with 3% FBS which should promote cell adhesion.

To determine whether mammosphere culture also triggers EMT, we examined the expression levels of markers associated with EMT. CD44^+^ cells generated by mammosphere culture consistently downregulated the expression of mRNAs encoding epithelial markers, such as E-cadherin, and upregulated mRNAs encoding mesenchymal markers, such as vimentin and twist, even at 12 h after culture in mammosphere media (Fig. 4C). These results suggest that CD44^+^ cells generated by mammosphere culture are also associated with EMT.

To determine further whether cytokines (EGF and bFGF) present in mammosphere culture medium promote EMT, and thus result in the enrichment of CD44^+^ cells, CD44^−^ cells were cultured in standard adherent culture conditions supplemented with 20 ng/ml EGF and 20 ng/ml bFGF. Similarly, cells exhibited a spindle-like morphology after 24 h culture in standard media containing EGF and bFGF (Fig. 4D-a). The significantly upregulated expression at mRNA levels of mesenchymal markers and CD44 was detectable, even at 12 h after culture in the media (Fig. 4D-b). Accordingly, CD44^+^ cells were significantly enriched after a 1-week culture, as shown by flow cytometric analysis (Fig. 4D-c). Conversely, when cultured in mammosphere media without EGF and bFGF, CD44^+^ cells were not enriched and the majority of CD44^−^ cells underwent apoptosis (data not shown). All of these results suggest that as in the enrichment of CD44^+^ cells by IL-6, the enrichment of CD44^+^ cells by mammosphere culture is the result of cytokine-mediated EMT by EGF and bFGF.

### EMT generally exists in mammosphere culture

**Figure 5:**
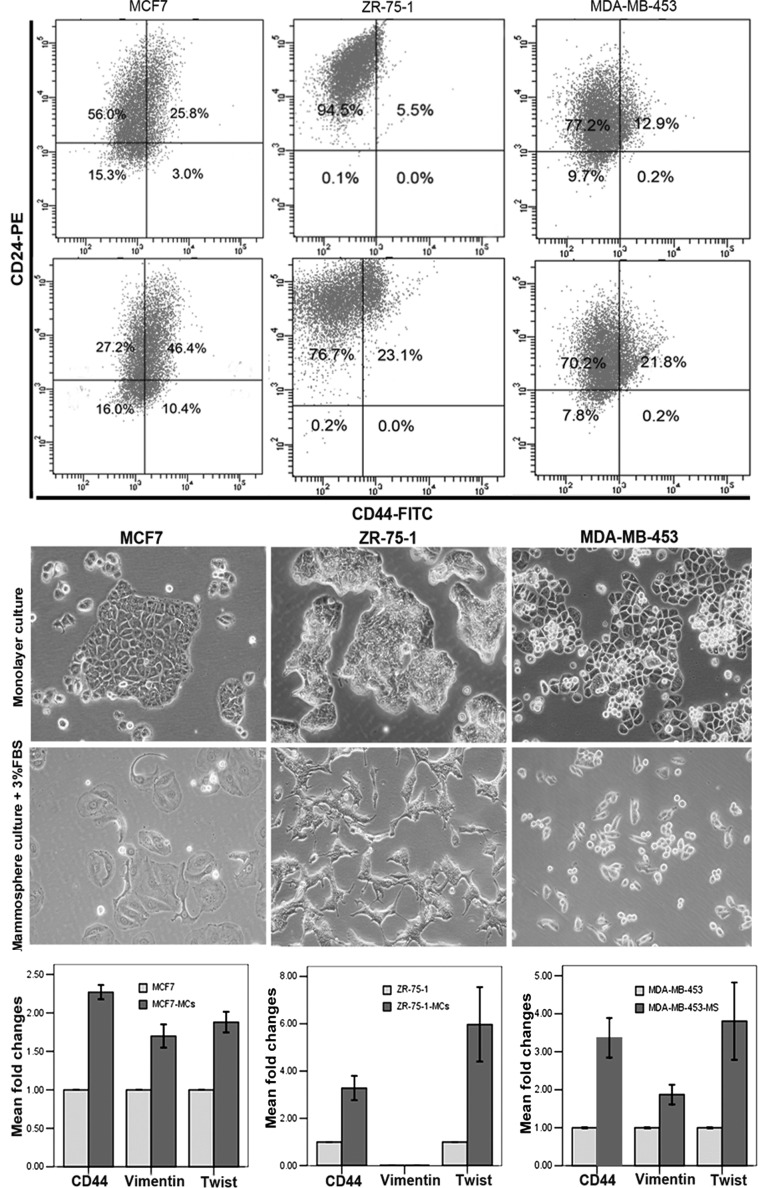
(A) Mammosphere culture of MCF7 significantly enriched cultures for CD44^+^CD24^−/low^ cells; mammosphere culture of ZR-75-1 or MDA-MB-453 cells enriched for CD44^+^ cells but not for CD44^+^CD24^−/low^ cells. (B) All three cell lines exhibited mesenchymal-like morphology after 2-day culture in mammosphere media supplemented with 3% FBS. (C) MCF7, ZR-75-1 and MDA-MB-453 cells developed a gene expression pattern consistent with EMT even within 12 h of mammosphere culture, including induction of mesenchymal markers, vimentin and twist, along with the induction of CD44.

To determine whether the EMT process occurs generally in mammosphere culture, we performed homologous experiments using three other breast cancer cell lines, MCF7, ZR-75-1, and MDA-MB-453. Mammosphere culture of MCF7 significantly enriched cultures for CD44^+^CD24^−/low^ cells (Fig. 5A), which was consistent with previous studies (17). However, as with T47D cells, mammosphere culture of ZR-75-1 or MDA-MB-453 cells enriched for CD44^+^ cells but not for CD44^+^CD24^−/low^ cells (Fig. 5A). All three cell lines exhibited mesenchymal-like morphology after a 2-day culture in mammosphere media supplemented with 3% FBS (Fig. 5B). As for T47D cells, MCF7, ZR-75-1 and MDA-MB-453 cells developed a gene expression pattern consistent with EMT even within 12 h of mammosphere culture, including induction of mesenchymal markers, vimentin and Twist, along with the induction of CD44 (Fig. 5C). These observations further support our conclusion that EMT promotes the enrichment of CSCs in the mammosphere culture system.

## Discussion

IL-6 is recognized as a major mediator involved in the regulation and maintenance of the inflammatory response. IL-6 is elevated in human breast tumors and breast cancer patient sera, and is associated with a poor prognosis in breast cancer (5). Almost all solid tumors are characterized by the presence of an inflammatory component in their microenvironments, including IL-6 (18), yet the link between IL-6 and BrCSCs remains poorly understood. We now show that the breast cancer cells T47D, are capable of producing CD44^+^ cells with stem-like properties on exposure to IL-6, which suggests that IL-6 promotes the induction of CSCs. CSCs are responsible for tumor initiation, sustaining tumor growth (19), tumor metastasis (20), and resistance to conventional anti-tumor therapies (15,21). These data explain, at least in part, why IL-6 is capable of triggering malignant features in human breast cancer cells.

EMT has been described over the past decade as a cellular biological program that is required for the remodeling of cells and tissues during embryogenesis, during certain types of wound healing, and during the acquisition of malignant traits by carcinoma cells (22,23). Many types of cancer cells, except for primary carcinomas, appear to rely on the EMT program to facilitate execution of most of the invasion-metastasis cascade (23). Mani *et al* (13) demonstrated for the first time that EMT induces properties of BrCSCs in addition to endowing cells with migratory and invasive potential. In this study, we have shown that IL-6 mediates the EMT process and promotes the generation of CD44^+^ cells with CSC-like attributes. This supports the important role of EMT in the generation of cancer stem-like cells.

Mammosphere culture has been used widely for the enrichment of mammary epithelial stem cells (24) and breast cancer stem cells (17). We examined whether mammosphere culture conditions ***per se*** induced EMT in the epithelial T47D breast cancer cell line. As anticipated, the mammosphere culture induced EMT within 12 h, involving the abrogation or induction of EMT-associated markers. Interestingly, when cultured in mammosphere media supplemented with 3% FBS, cells consistently displayed a mesenchymal-like morphology. We also showed here that EGF and bFGF, two important cytokines present in mammosphere media, consistently induced EMT, showing a spindle-like morphology and induction of mesenchymal markers, which was concomitant with the enrichment of CD44^+^ cells. Conversely, without EGF and bFGF, CD44^+^ cells could not been enriched and a majority of cells underwent apoptosis in mammosphere media. It seems to be apparent that the enrichment of CD44^+^ cells by mammosphere culture occurs as a result of cytokine-mediated EMT with EGF and bFGF, and is analogous to the enrichment of CD44^+^ cells by IL-6 exposure. Using other three epithelial-like breast cancer cell lines, we further confirmed that this phenomenon generally existed in mammosphere culture. Indeed, a similar connection between EGF or bFGF and EMT was previously shown in human breast carcinoma cell line, PMC42-LA (25), and tubular cell lines (26).

Additionally, it was thought that CSCs survive, self-renew, and propagate in mammosphere media, whereas non-stem cancer cells undergo cell death, which accordingly leads to the enrichment of CSCs. In contrast, we showed that mammosphere culture of CD44^−^ cells enriched for CD44^+^ cancer stem-like cells through an EMT process. The data challenge the traditional view of mammosphere culture as an *in vitro* surrogate assay of self-renewal capacity. In view of these results, we suggest that EMT is an important mechanism for the induction of cancer cells with stem-like properties.

Collectively, the findings presented here describe a link between IL-6 and BrCSCs, and an important mechanism of CSC-formation in mammosphere culture. These findings demonstrate that induction of EMT in differentiated breast epithelial tumor cells is sufficient to generate a subpopulation of cancer cells with stem cell characteristics. Thus, EMT is not only important for cells to escape from the immediate vicinity of the tumor, but may also sustain primary tumor growth as well as promoting the initiation and establishment of secondary tumors.
